# Retinal layer parcellation of optical coherence tomography images: Data resource for multiple sclerosis and healthy controls

**DOI:** 10.1016/j.dib.2018.12.073

**Published:** 2018-12-28

**Authors:** Yufan He, Aaron Carass, Sharon D. Solomon, Shiv Saidha, Peter A. Calabresi, Jerry L. Prince

**Affiliations:** a Dept. of Electrical and Computer Engineering, The Johns Hopkins University, Baltimore, MD 21218, USA; b Dept. of Neurology, The Johns Hopkins School of Medicine, Baltimore, MD 21287, USA; c Wilmer Eye Institute, The Johns Hopkins School of Medicine, Baltimore, MD 21287, USA

## Abstract

This paper presents optical coherence tomography (OCT) images of the human retina and manual delineations of eight retinal layers. The data includes 35 human retina scans acquired on a Spectralis OCT system (Heidelberg Engineering, Heidelberg, Germany), 14 of which are healthy controls (HC) and 21 have a diagnosis of multiple sclerosis (MS). The provided data includes manually delineation of eight retina layers, which were independently reviewed and edited. The data presented in this article was used to validate automatic segmentation algorithms (Lang et al., 2013).

**Specifications table**

**Table:** 

Subject area	*Ophthalmology*
More specific subject area	*Human retina, Multiple sclerosis*
Type of data	*Optical coherence tomography*
How data was acquired	*Spectral Domain OCT using Spectralis OCT system*
Data format	*Raw and Processed*
Experimental factors	*Human retina without pretreatment*
Experimental features	*The structure of the human retina was examined with SD-OCT*
Data source location	*The Johns Hopkins Hospital, Baltimore, MD 21287 USA*
Data accessibility	*Public download*
Related research article	*A. Lang, A. Carass, M. Hauser, E.S. Sotirchos, P.A. Calabresi, H.S. Ying, and J.L. Prince, “Retinal layer segmentation of macular OCT images using boundary classification”, Biomedical Optics Express, 4(7):1133–1152.*

**Value of the data**

• This is currently the largest public data set of manually delineated layers of the human retina from OCT scans.
• The data are fully delineated so thickness analysis and algorithm comparison can be performed.
• The data can also be used for training and validation of segmentation algorithms.

## 1 Data

**Fig. 1:**
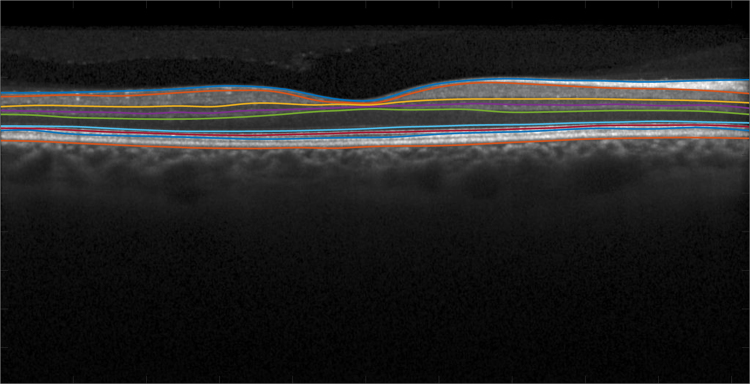
An example B-scan images, showing the included manual delineations.

The data presented in this article was used to validate automatic segmentation algorithms [1], [2], [3]. The data comprise scans of the right eye of 35 subjects scanned on a Spectralis OCT system (Heidelberg Engineering, Heidelberg, Germany). The cohort comprises 14 healthy controls (HC) and 21 patients with multiple sclerosis (MS); complete demographic information is included in Table 1. All the scans were manually delineated once using internally developed software. For each subject, we provide a retinal OCT image consisting of 49 B-scans and 9 layer boundaries delineated in every B-scan. An example B-scan and the manual delineation is shown in Fig. 1. The provided layers are listed in Table 2. The data is available for download from: http://iacl.jhu.edu/Resources.

**Table 1:** Demographic details for the data. The top line is the information of the entire data set, subsequent lines are specific to that category. *N* (M/F) denotes the number of patients and the male/female ratio, respectively. Age is the mean age (and standard deviation) in years at scan time. The key for the table is: HC – Healthy Controls; MS – Multiple Sclerosis.

**Dataset**	***N* (M/F)**	**Age Mean (SD)**
ALL	35 (6/29)	39.49 (10.94)
HC	14 (2/12)	35.77 (13.03)
MS	21 (4/17)	41.97 (8.77)

**Table 2:** Manually delineated retina layers.

Layer name	*Abbreviation*
Retina nerve fiber layer	*RNFL*
*Ganglion cell layer and inner plexiform layer*	GCL+IPL
*Inner nuclear layer*	*INL*
*Outer plexiform layer*	*OPL*
*Outer nuclear layer*	*ONL*
*Inner photoreceptor segments*	*IS*
*Outer photoreceptor segments*	*OS*
*Retinal pigment epithelium*	*RPE*

## 2 Experimental design, materials, and methods

The Spectralis scanner׳s automatic real-time function is used to acquire the scans. Each B-scan was averaged at least 12 images at the same location and the signal-to-noise ratio of the final averaged scans was at least 20 dB. A macular cube scan (20° × 20°) was acquired with 49 B-scans, each B-scan consists of 1024 A-scans, and each A-scan has 496 pixels. The B-scan resolution varied slightly between subjects, the lateral resolution (between A-scans) has a mean over all the subjects of 5.8 µm (±0.2) and the axial resolution (between two pixels in an A-scan) is 3.9 µm (±0.0). The through-plane distance (slice separation) has a mean of 123.6 µm (±3.6) between images, resulting in an imaging area of approximately 6 × 6 mm^2^. The volume data was exported from the scanner using the.vol file format. To facilitate data use, we also provide scripts for reading the.vol files into Matlab. The manual delineations are saved in the.mat file which can be directly imported into Matlab.

Automatic segmentation methods evaluated using this dataset can be found in Refs. [1], [2], [3], [4], [5], [6] and other works using this data, in whole or part, include [7], [8], [9], [10], [11], [12].
