# Supplementary material for: Retinal layer parcellation of optical coherence tomography images: Data resource for multiple sclerosis and healthy controls
Source: Data Brief. 2018 Dec 28;22:601–4. doi: 10.1016/j.dib.2018.12.073 (PMC6327073; doi:10.1016/j.dib.2018.12.073)
Supplement: Supplementary file 1 — Supplementary material [file mmc1.pdf]

JOHNS HOPKINS
WHITING SCHOOL
of ENGINEERING

Department of Electrical and
Computer Engineering,
Whiting School of Engineering,
105 Barton Hall / 3400 N. Charles St.,
Baltimore, MD 21218-2686
410-516-7031 / Fax 410-516-5566
http://www.ece-jhu.org/

All undersigned authors certify that they have NO affiliations with or involvement in any organization or entity with any financial interest (such as honoraria; educational grants; participation in speakers' bureaus; membership, employment, consultancies, stock ownership, or other equity interest; and expert testimony or patent-licensing arrangements), or non-financial interest (such as personal or professional relationships, affiliations, knowledge or beliefs) in the subject matter or materials discussed in this manuscript.

YH, AC, SDS, SS, JLP.

The following authors have declarations:

PAC has received personal consulting fees for serving on SABs for Biogen and Disarm Therapeutics. He is PI on grants to JHU from Biogen, Novartis, Sanofi, Annexon, and MedImmune.

RTS has received personal consulting fees from Genentech/Roche.
